# Protocol: The Impact of Parental Incarceration on Families Affected: An Evidence and Gap Map: A Systematic Review

**DOI:** 10.1002/cl2.70055

**Published:** 2025-08-17

**Authors:** Daragh Bradshaw, Lynn Fenton, Fiona Donson, Aisling Parkes, Ben Raikes, Leonie Ludwig, Julie Poehlmann

**Affiliations:** ^1^ University of Limerick Limerick Ireland; ^2^ University College Cork Cork Ireland; ^3^ University of Huddersfield Huddersfield UK; ^4^ University of Madison‐Wisconsin Madison Wisconsin USA

## Abstract

This is the protocol for a Campbell systematic review. The objectives are as follows. Identify and map all existing primary studies and systematic reviews (published and unpublished) on social, emotional, educational, and behavioural issues in families affected by parental incarceration, creating a live, searchable and publicly available Evidence and Gap Map. We will focus on children affected by parental incarceration, their caregivers, and incarcerated parents. This map will include primary studies, reviews as well as intervention and evaluation papers. The purpose of the current research is to provide researchers, practitioners, and policymakers with a map of available evidence, identifying areas that warrant additional research or synthesis, as well as highlighting gaps in our knowledge.

## 1 Background

### 1.1 The Problem, Condition or Issue

A growing literature demonstrates the potentially devastating impact that incarceration can have on entire families. We define the family in an exclusive sense of immediate caregivers, children, and incarcerated parents. The family can be impacted across multiple outcomes for extended periods of time. Social inequities include homelessness and poverty due to loss of income and employment or loss of child support payment (Muentner et al. 2019), or trouble succeeding in school (Cho 2010). Others experience emotional problems connected to low self‐esteem, anxiety and depression (Wildeman and Turney 2014; Foster and Hagan 2013; Lee et al. 2013), and many experience future behavioural issues (Besemer et al. 2017). Similarly, the caregivers of children with parents in prison report increased levels of physical and mental health difficulties (Chui 2016; Bradshaw and Muldoon 2020).

Worldwide trends in prison populations have altered the experience of incarceration from a marginal to an ordinary phenomenon (Pattillo et al. 2004). A consequence of this rise is that families are increasingly being impacted, with between 45% and 87% of prisoners reported as being parents (Glaze and Maruschak 2008), with an estimated 7% of children in the US have experienced parental incarceration (Provencher and Conway 2019) and a further 2.1 million children in the council of Europe countries (Philbrick et al. 2014). Additionally, non‐incarcerated residents living in communities with high incarceration rates are at an increased risk of negative physical, psychological and educational outcomes (Haskins et al. 2018; Hagan and Foster 2012).

However, it is important to acknowledge that the experience of parental incarceration is not equal (Haskins et al. 2018) or homogeneous (Adalist‐Estrin 2018). For example, research demonstrates that in the context of maternal rather than paternal incarceration, children are more likely to be exposed to increased risk of negative outcomes (Dallaire 2007), and school dropout (Cho 2010). Maternal incarceration is usually far more disruptive for children than paternal incarceration. In the United Kingdom, only 9% of children with mothers in prison remain in the home they lived in before their mother's incarceration, compared to 95% remaining in the same home if their father is imprisoned (Home Office 2007). This often results in a change of school and moving away from support networks (Minson 2017), including siblings at the time when they need them and stability the most. Conversely, Poehlmann‐Tynan and Turney (2021) suggest that findings are mixed for internalising problems in the context of maternal but not paternal incarceration. This disparity may be related to timing and risk factors associated with the incarcerated mothers themselves (Wildeman and Turney 2014). Relatedly, Murphey and Cooper (2015) found that in the United States, the experience of parental incarceration is highest among young, poor, and rural children. This is reflected in other contexts, for example, Lalor (2017) found that that while the minority ethnic identity of Traveller is estimated to be 0.06% of the general population in an Irish context, 15% of the male and 22% of the female prison population identify as Traveller. Additionally, Johnson et al. (2024) found that while parental incarceration contributed to an increased risk of negative health outcomes among racialised groups, it depended upon the nature of the outcomes and context in which it occurred.

Consequently, it is important to consider the wider aspects of the experience of parental incarceration when exploring the overall effect. For example, previous research highlights the impact of witnessing parent arrest, criminal activity, and sentencing (Metcalfe et al. 2023; Dallaire and Wilson 2010); the frequency and length of incarceration (Andersen 2016); available interventions and support for the impact of incarceration (Eddy and Burraston 2018); subsequent caregiving environment (Arditti and Johnson 2022; Davis and Shlafer 2017); frequency and type of parent‐child communication or visits (Poehlmann et al. 2010); re‐entry/recidivism issues (Eddy and Burraston 2018). Additionally, the impact of the parental separation will be impacted by the age and developmental profile of the child (Poehlmann et al. 2010).

### 1.2 The Intervention

### 1.3 Why It Is Important to Develop the Evidence and Gap Map (EGM)

In recognition of the various threats to outcomes related to Social, Emotional, and Behavioural outcomes for children and families affected, there are a growing number of initiatives specifically focused on supporting those at risk (Eddy and Burraston 2018). These initiatives use a variety of different approaches, such as enhanced visiting schemes, homework clubs, parenting programmes, and can focus on the individual, family, community or school‐based interventions (e.g., McLaughlin et al. 2016; Roberts and Loucks 2015).

However, effective initiatives need to be grounded on evidence and, in particular, on research that is co‐built with communities. While there has been a marked increase in research in this area, it covers a wide range of topics in an equally diverse number of jurisdictions and disciplines with little or no links between approaches (Wildeman et al. 2018). Consequently, evidence is often scattered around different databases, websites and the grey literature, and is often presented in inaccessible formats. This can result in agencies and researchers implementing interventions without a sufficient evidence base or duplicating efforts by investing in interventions and studies already developed elsewhere. Lack of overview of the existing evidence undermines the ability of agencies tasked with supporting families to develop evidence‐based supports, particularly for agencies with limited funds (Snilstveit et al. 2016). This has resulted in a call for a more coordinated approach between researchers, NGO's and policymakers (Roberts and Loucks 2015).

There are a number of literature and systematic reviews available mainly focusing on intervention studies (e.g., but not limited to; Paynter et al. 2020; Bradshaw and Muldoon 2017; Tremblay and Sutherland 2017; Armstrong et al. 2017; Purvis 2013; Buston et al. 2012; Newman et al. 2011; Loper and Novero 2010), as well as child outcomes such as health and well‐being (e.g., but not limited to: Wildeman et al. 2018; Boch and Ford 2018; Turney and Haskins 2019; Whitten et al. 2019; Poehlmann‐Tynan and Turney 2021; Austin et al. 2022; Riyantono et al. 2022; Luk et al. 2023), behavioural outcomes such as anti‐social behaviour or externalising issues (e.g., but not limited to: Murray et al. 2012; Besemer et al. 2017; Whitten et al. 2019) educational outcomes (e.g., Murray et al. 2012; Luk et al. 2023; Haskins et al. 2018), coping styles (Tremblay and Sutherland 2017), as well as reviews on the wider family (e.g., Wildeman and Lee 2021; Lee and Wildeman 2021).

However, many of these reviews either do not include a systematic approach or include a narrow focus of interest on specific child‐related outcomes. With no review capturing the impact on caregiver outcomes or incarcerated parent outcomes. Additionally, these reviews are predominantly focused on US‐based samples (see Luk et al. 2023 and Eddy and Burraston 2018 for exceptions) with limited stakeholder involvement.

Taken together, this demonstrates the need to ‘map’ the available evidence and the gaps in evidence in a format that is useful and accessible to the public and particularly agencies tasked with providing support to those in need. An EGM differs to other systematic reviews in a number of ways: (1) It is broader in focus and can include more studies. (2) It provides a visual matrix enabling ease of access for practitioners and researchers. (3) It explicitly collaborates with existing agencies to ensure findings are relevant to practitioners as well as academic needs. (4) There is a specific focus on identifying available research as well as gaps in the evidence base. Finally (5), EGMs are living repositories of evidence more easily accessed by practitioners and are easily revised and updated to accommodate emerging research.

The proposed EGM asks the question, what is the impact of parental incarceration for families affected? We will be looking specifically at the impact of parental incarceration as well as different aspects impacting outcomes. It is being undertaken by the Centre for Social Issues Research at the University of Limerick, together with researchers from the University College Cork and the University of Wisconsin‐Madison. It has been developed in partnership with key stakeholders, practitioners and academics in the area. The benefits of an EGM include:

1. Funders can quickly assess the areas where there is already a saturation of evidence, see where the knowledge gaps lie, and direct much‐needed resources toward those areas;
2. Policymakers can access the map to see where evidence already exists to inform policy and practice;
3. Members of the public, including parents, carers, and the young people themselves, can quickly access information that is of relevance (and of interest) to them;
4. Researchers can minimise research waste that occurs due to duplication of effort (Keenan et al. Unpublished grant proposal, 14th October 2020).

While Jackson et al. (2019) conducted an EGM identifying empirical evidence on outcomes of security and justice interventions, the team has been unable to locate any EGMs that focus specifically on the Social, Emotional, and Behavioural Problems in families affected by parental incarceration.

## 2 Objectives

Identify and map all existing primary studies and systematic reviews (published and unpublished) on social, emotional, educational and behavioural issues in families affected by parental incarceration, creating a live, searchable and publicly available EGM. We will focus on children affected by parental incarceration, their caregivers, and incarcerated parents. This map will include primary studies, reviews as well as intervention and evaluation papers. The purpose of the current research is to provide researchers, practitioners, and policymakers with a map of available evidence, identifying areas that warrant additional research or synthesis, as well as highlighting gaps in our knowledge.

## 3 Methods

### 3.1 EGM: Definition and Purpose

An EGM is a systematic approach to identify and report on the research activity in a specific domain. It outlines the nature, characteristics and volume of research available. This information can be presented on a graphical display involving a matrix of intervention categories in rows with outcome domains in columns. Additional visual prompts can be then used to indicate further information, such as study design, location, and quality of evidence. Funding and future research can then be directed to areas where they can have the greatest impact.

Drawing on previous EGMs (Cáceres‐Escobar et al. 2020), this project will involve seven steps:

1. Scoping and development of the EGM framework;
2. Systematic and comprehensive search;
3. Eligibility screening;
4. Data extraction;
5. Quality appraisal;
6. Analysis;
7. Maintenance and updating the map.

### 3.2 Framework Development and Scope

To develop a framework that best represents research on parental incarceration, the framework will be developed in consultation with key stakeholders (see below for list of key stakeholders involved in this project). This framework will form the basis for the systematic search, the screening and data extraction, and visual presentation of the included evidence.

We will follow an EGM framework as a matrix with rows containing population of interest and columns containing the relevant outcomes. The map will also include information filters such as the theoretical perspective, study design, nature of the incarceration (e.g. Jail/prison/open prison), gender (Maternal v paternal incarceration) as well as geographical context.

### 3.3 Stakeholder Engagement

The proposed research has arisen as a consequence of a collaboration between the Irish Penal Reform Trust (IPRT), the Action for Children and Families of Prisoners Network (CFPN) and researchers from the University of Limerick and the University College Cork.

Together, the IPRT and the CFPN comprise people with experience of familial imprisonment, community‐based organisations, researchers, academics and advocacy groups with a strong interest in advancing the rights and needs of children and families with loved ones in prison.^1^ Their shared aim is to bring about positive, sustained change for children and families affected by imprisonment and advocate for national policy change.

An Advisory Board for the EGM will be established, and membership will be drawn from the stakeholders represented in our national network as well as consultation with the International Coalition of Children with Incarcerated Parents. INCCIP Board and Members, https://inccip.org/about-us/, include researchers, practitioners and advocates across five continents, providing this EGM with a truly global reach. The Advisory Board will meet virtually and will be consulted at all stages of the EGM, contributing to defining its scope and developing the population outcome framework that will be used to construct the map.

This framework will include items such as incarceration characteristics (for example, nature of the incarceration [remand, jail or prison], length of incarceration, dosage, frequency, age, gender, ethnicity, access to visits), availability of prison‐related family supports and relevant outcomes. The map will also include information about the theoretical perspective, study design, and geographical/jurisdictional context.

This framework will be finalised in consultation with the stakeholder advisory board.

### 3.4 Conceptual Framework

There are many mechanisms through which disadvantages caused by parental incarceration can be transmitted, including but not limited to attachment or identity perspectives, with stigma being particularly significant.

Poehlmann and Turney (2021) emphasised how developmental perspectives are key to understanding the experience and impact of parental incarceration for both caregivers and children affected, such as timing, duration and frequency of parental incarceration, processes underlining the experience, potential developmental cascades, as well as mediators and moderators of any effects. For example, Haskins (2015) argues that early childhood represents a critical period in a child's emotional, behavioural and social development and can be negatively impacted by PI. Specifically, parental responsivity and sensitivity to a child's needs enhance positive PCG‐child relationships (Bakermans‐Kranenburg et al. 2003). However, as the child develops through adolescence, a different array of needs and outcomes becomes relevant.

Relatedly, Arditti (2016) argues that the impact on the child and their caregivers does not occur in a vacuum and can be understood through a stress‐proximal model. Specifically, incarceration impacts the process and relationships between children and other family members. For example, contextual stressors associated with the imprisonment of a partner, such as economic, social and psychological issues, undermine PCG's well‐being. Reflecting this, Bradshaw et al. (2020) demonstrate that PI negatively impacts PCG depression which in turn undermines the PCG‐child relationship and increases future levels of internalising and externalising difficulties for children who have experienced PI.

Equally, the impact of PI on families affected can be understood using an Identity perspective. Within this context, identity theory has increasingly been recognised as central to understanding the association of PI and family outcomes (Asencio and Burke 2011). Labelling theory suggests that social expectations can lead to self‐fulfilling prophecies (Murray et al. 2014). This can have real implications for incarcerated fathers and their families (Besemer et al. 2017). For example, once an individual acquires a particular identity label, such as delinquent, the individual can foster this self‐image and amplify identity‐conforming anti‐social behaviours (Farrington and Murrray 2014; Besemer et al. 2017). This can strengthen social ties with deviant groups (Bernburg et al. 2006).

Goffman (1963) described how discrimination and prejudice harms not only the individual with the stigmatised identity, but also extends to their family and associates. For example, family members of people with stigmatised identities reported experiences of shame and culpability as well as strained and distant relationships with others (Östman and Kjellin 2002; Corrigan and Miller 2004). Codd (2013) describes how incarceration can be understood as a ‘courtesy stigma’ (Goffman 1963), where the family shares the spoiled identity of the inmate. Corrigan et al. (2006) found that families of those with an anti‐social behaviour identity, such as drug addiction, faced an increased risk of being blamed and socially shunned. Compounding this, there is converging evidence to suggest that once a family is characterised as a ‘criminal family’, they receive more attention and differential treatment from others (Theobald et al. 2016). For example, families of known offenders are more likely to be processed through the courts after an offence (Besemer et al. 2017), as well as face reduced access to jobs, housing and education (Bernburg et al. 2006; Hagan and Foster 2012). Dallaire et al. (2010) found that teachers expected reduced academic ability in children affected by PI rather than in children from single‐parent families not affected by PI. Moreover, this situation is aggravated when the treatment is perceived to be unfair by those affected. This can lead to anger and defiance and can increase anti‐social behaviour and mental health difficulties (Giordano 2010). Conversely, Bradshaw et al. (2024) demonstrate that experiences of parental incarceration at the age of 9 can undermine positive social group membership in early adolescence. Reduced social group membership can have a long‐term impact on both biomarkers of health in the general population (McMahon et al. 2024) as well as prosocial behaviours in children specifically impacted by parental incarceration (Bradshaw et al. 2024).

### 3.5 Dimensions

To develop a framework that best represents research on parental incarceration, the framework will be developed in consultation with key stakeholders (see Section 3.3 for a list of key stakeholders involved in this project). This framework will form the basis for the systematic search, the screening and data extraction, and the visual presentation of the included evidence.

We will follow a population‐by‐outcome EGM framework as a matrix with rows containing the population of interest and columns containing the relevant outcomes. The map will also include information filters such as the theoretical perspective, study design and geographical context.

The framework for the EGM informs the inclusion and exclusion criteria. This framework includes primary dimensions of outcomes, broken down by child, parents, caregiver and community context (see Table 1).

**Table 1:** Framework dimensions.

	Health	Educational	Social outcomes	Emotional outcomes	Behavioural outcomes
Child					
Incarcerated parent					
Primary caregiver					

#### 3.5.1 Types of Study Design

This EGM will map all available research, including qualitative and quantitative designs as well as systematic reviews and meta‐analyses. Specifically, we will include:

- Cross‐sectional and longitudinal data reporting the prevalence of specified outcomes.
- Cohort studies (prospective and retrospective) where the individuals are identified and followed up over time to explore the impact on specified outcomes.
- Case‐control studies where individuals who have experienced parental incarceration are compared with individuals who have not.

Case studies or series focusing on small groups of individuals or families who have experienced parental incarceration.

#### 3.5.2 Types of Intervention/Problem

Empirical studies and reviews focusing on the impact of parental incarceration.

However, studies including a measure of parental incarceration as part of a list of adverse childhood experiences but do not isolate the unique impact of PI will be excluded.

#### 3.5.3 Types of Population (as Applicable)

Families impacted by parental incarceration. This includes children, their incarcerated parents, and their caregivers. We will include studies of families after parents have been released from prison and during incarceration. We will not be including studies that only focus on the impact of involvement with the justice and policing systems during the trial.

#### 3.5.4 Types of Outcome Measures (as Applicable)

Core outcomes were decided through consultation with the stakeholder advisory board and will include the following:

- Child development, for example:
  ∘ Physical Health
  ∘ Mental Health
  ∘ Behavioural
  ∘ Educational
  ∘ Social
  ∘ Attachment
  ∘ Eating and food security
  ∘ Stigma
  ∘ Institutional care
  ∘ Housing and homelessness
  ∘ Caregiving experiences
- Incarcerated Parent outcomes, for example:
  ∘ Family relationships
  ∘ Mental health
  ∘ Physical health
  ∘ Financial/economic security
  ∘ Parenting efficacy/confidence
  ∘ Social connection
  ∘ Stigma
  ∘ Institutional supports
  ∘ Homelessness
- Caregiver outcomes, for example:
  ∘ Family relationships
  ∘ Stress
  ∘ Efficacy/confidence
  ∘ Mental health
  ∘ Social Connections
  ∘ Financial implications

Outcomes listed will not be considered as exclusion criteria.

### 3.6 Other Eligibility Criteria

The inclusion and exclusion criteria will be determined on three eligibility questions:

a. Does the paper report on any of the following types of designs
  1. Cross‐sectional and longitudinal data reporting the prevalence of specified outcomes.
  2. Cohort studies (prospective and retrospective) where the individuals are identified and followed up over time to explore impact on specified outcomes.
  3. Case‐control studies where individuals who have experienced parental incarceration are compared with individuals who have not.
  4. Case studies or series focusing on small groups.
b. Does this study include child, parent or family outcomes?
c. Is parental incarceration a standalone measure and not merged in a list of total stressful life events?

#### 3.6.1 Types of Location/Situation (as Applicable)

#### 3.6.2 Types of Settings (as Applicable)

Any context that addresses the impact of Parental incarceration.

### 3.7 Search Methods and Sources

Search strategy has been developed with the guidance of a Campbell information retrieval specialist (CK) and will be conducted and reported following Campbell Collaboration guidelines (White et al. 2020).

The search strategy has been built around three concepts of (1) setting of interest (terms relating to incarceration such as Prison, incarceration, jail, correctional facility), (2) population of interest (terms relating to children, parents, families and caregivers) and (3) terms relating to outcomes (such as emotional, behavioural and social issues). An example of the search string has been piloted in PsycArticles (Ebsco) (see Table 2).

**Table 2:** APA psych articles search conducted on 9 May 2022.

Step	Search fields	Search syntax	
1	Title	(child* OR adolesce* OR teen* OR youth* OR young* OR minor* OR kid* OR student* OR school* OR pube* OR paren* OR pater* OR father* OR dad* OR mater* OR mother* OR mum* OR mom* OR pregn* OR famil* OR son* OR daughter*)	37,564
2	Abstract	(child* OR adolesce* OR teen* OR youth* OR young* OR minor* OR kid* OR student* OR school* OR pube* OR paren* OR pater* OR father* OR dad* OR mater* OR mother* OR mum* OR mom* OR pregn* OR famil* OR son* OR daughter*)	87,558
3	Keywords	(child* OR adolesce* OR teen* OR youth* OR young* OR minor* OR kid* OR student* OR school* OR pube* OR paren* OR pater* OR father* OR dad* OR mater* OR mother* OR mum* OR mom* OR pregn* OR famil* OR son* OR daughter*)	52,710
4	Subject or indexing terms	(child* OR adolesce* OR teen* OR youth* OR young* OR minor* OR kid* OR student* OR school* OR pube* OR paren* OR pater* OR father* OR dad* OR mater* OR mother* OR mum* OR mom* OR pregn* OR famil* OR son* OR daughter*)	53,323
5	—	1 OR 2 OR 3 OR 4	110,174
6	Title	prison* OR incarcerat* OR imprison* OR jail* OR gaol* OR correction* OR offend* OR detain*	4593
7	Abstract	prison* OR incarcerat* OR imprison* OR jail* OR gaol* OR correction* OR offend* OR detain*	5355
8	Keywords	prison* OR incarcerat* OR imprison* OR jail* OR gaol* OR correction* OR offend* OR detain*	1763
9	Subject or indexing terms	prison* OR incarcerat* OR imprison* OR jail* OR gaol* OR correction* OR offend* OR detain*	2573
10	—	6 OR 7 OR 8 OR 9	8045
11	—	5 AND 10	3816

#### 3.7.1 Electronic Databases

Studies will be identified through a systematic search of electronic databases, grey literature and contacting key authors and organisations in the area. Based on the University of Limerick database subscriptions, we will search the following key databases:

- British Education Index (EBSCOhost)
- Child Development & Adolescent Studies (EBSCOhost)
- Campbell Collaboration Library
- Cochrane Central Register of Controlled Trials
- CINAHL Plus with Full Text (EBSCOhost)
- Criminal Justice Abstracts*
- Criminal Justice*
- Dissertation & Theses Global* (ProQuest)
- Educational Administration Abstracts (EBSCOhost)
- ERIC (EBSCOhost)
- International Bibliography of the Social Sciences (IBSS) (ProQuest)
- MEDLINE (Ovid)
- National Criminal Justice Reference Service*
- PsycINFO (1806–present) (Ovid)
- Scopus
- Social Sciences Citation Index (Web of Science Core Collection)
- The National Resource Centre on Children and Families of the Incarcerated*

World Prison Brief website (Research and Publications section, http://www.prisonstudies.org/research-publications/intro)*

- https://njlaw.rutgers.edu/cj/gray/index.php*
- crimesolutions.gov*
- Home Office/MOJ UK*
- Swedish National Council on Crime Prevention (Brå)*

*indicates grey literature.

#### 3.7.2 Searching Other Resources

We also intend to conduct a search of reference lists of previous reviews and eligible articles to identify any additional studies not identified through the electronic search. Authors of selected studies, as well as experts in the area, will be contacted to identify unpublished or impending studies that may be included in this review. This stage will be particularly relevant to identifying qualitative studies as systematic review searches often do not provide a comprehensively identified relevant qualitative research (Greenhalgh and Peacock 2005).

### 3.8 Analysis and Presentation

#### 3.8.1 Report Structure

A population‐outcome framework will be used including primary studies or systematic reviews.

#### 3.8.2 Filters for Presentation

In addition to the outcomes listed above, the map will include the following filters:

1. Country or geographical region.
2. Gender of the incarcerated parent.
3. Nature of the incarceration (remand, jail or prison).
4. Ethnicity.
5. Theoretical perspective.
6. Quality of study.
7. Study design.

This framework will be finalised in consultation with the stakeholder advisory board.

#### 3.8.3 Dependency

This EGM will include primary studies, or systematic reviews. Where there are multiple reports relating to one study, these will be considered as a single study. Where a single report includes multiple studies, each study will be included on the map separately.

### 3.9 Data Collection and Analysis

#### 3.9.1 Screening and Study Selection

On completion of the systematic search, all studies will be imported into a bibliographic reference manager system, where duplicates will be removed. Title and abstract screening will be completed independently by two study authors using the inclusion/exclusion criteria listed above. Disagreements will be resolved through discussion. If a resolution cannot be achieved, disagreements will be resolved through discussion with a third author.

Full text of eligible documents will be downloaded and reviewed using the same procedure as outlined above.

#### 3.9.2 Data Extraction and Management

Study characteristics as well as the relevant outcomes listed above will be extracted from eligible studies will be extracted by two authors independently. Disagreements will be resolved through discussion and with arbitration by a third author if necessary. Coding sheets will be pretested before full data extraction (see Table 3, Appendix S1 for pilot coding sheet). In the event of missing data, the authors will contact the study authors.

**Table 3:** Pilot coding sheet.

	Yes	No
Eligibility criteria		
Is the paper a qualitative or quantitative empirical study or systematic review relating to parental incarceration?		
Does this study include child, parent or family outcomes?		
Is parental incarceration a standalone measure and not included in a list of other stressful life events?		
Did this study specify the gender of the incarcerated parent?		
Male		
Female		
Both		
Other		
Please specify		
Did this study specify the ethnicity of the incarcerated parent? (Please specify)		
Did this study indicate the gender of the study child?		
Male		
Female		
Both		
Other		
Please specify		
Publication type		
Empirical paper qualitative		
Empirical paper qualitative		
Study design		
Systematic review		
Intervention (please specify)		
Cross‐sectional study		
Longitudinal study		
Did this study specify the country or region where the incarceration took place?		
Please specify		
Quality/risk of Bias of evidence		
Cochrane Risk of Bias Tool 1		
AMSTAR 2		
ROBINS‐E		
Mixed Methods Appraisal Tool (MMAT)		
Did the study state the nature of the incarceration (remand, jail or prison)?		
Remand		
Jail		
Prison		
Did this study specify the length of incarceration?		
Please specify		
Did this study indicate a theoretical perspective?		
Please specify		
Does this study report child development outcomes?		
Physical health		
Mental health		
Behavioural		
Educational		
Social		
Attachment		
Eating and food security		
Stigma		
Institutional care		
Housing and homelessness		
Caregiving		
Other		
Please specify		
Does this study report any incarcerated parental outcomes?		
Mental health		
Physical health		
Parent identity		
Parenting behaviour		
Parenting knowledge		
Parenting attitude		
Other		
Please specify		
Does this study report parental (other than the incarcerated parent) outcomes?		
Mental health		
Physical health		
Financial/economic security		
Parenting efficacy/confidence/behaviour		
Social connection		
Stigma		
Institutional supports		
Homelessness		
Other		
Please specify		
Does this study report caregiver (other than biological parent) outcomes?		
Stress		
Efficacy/confidence		
Mental health		
Families, for example:		
Family relationships		
Does this study report community‐based outcomes?		
Other?		
Please specify		

#### 3.9.3 Tools for Assessing Risk of Bias/Study Quality of Included Reviews

All systematic reviews will be appraised in duplicate for quality using AMSTAR2 (Shea et al. 2017).

Primary studies will be assessed using the Cochrane Risk of Bias tool (version 2, Sterne et al. 2019).

ROBINS‐E Risk of Bias tool for non‐randomised studies experimental studies (Higgins et al. 2024).

For qualitative and mixed‐method studies, we will use the Mixed Methods Appraisal Tool (MMAT) (Hong et al. 2018).

#### 3.9.4 Methods for Mapping

The map will be produced using the EPPI Centre's Mapping Software (EPPI) (EPPI‐Mapper).

## Author Contributions

- Content: Daragh Bradshaw, Fiona Donson, Aisling Parkes, Julie Poehlmann and Ben Raikes.
- EGM methods: Daragh Bradshaw and Lynn Fenton.
- Statistical analysis: Daragh Bradshaw, Fiona Donson, Aisling Parkes, Ben Raikes and Lynn Fenton.
- Information retrieval: Lynn Fenton and Leonie Ludwig.

## Conflicts of Interest

It is likely that some co‐authors and members of the network will have authored studies that are included in the map. Consequently, data collection, screening, and quality assessment will be conducted by independent members of the Team.

## Preliminary Timeframe

- Date you plan to submit a draft protocol: May 2025.
- Date you plan to submit a draft EGM: May 2026.

## Plans for Updating the EGM

Review authors will update the map every 2 years.

## Sources of Support

### External Sources

Research is funded by a Family Matters Award from the St Stephens Green Trust, as well as a New Foundations Award from the Irish Research Council. The corresponding Author also received an Evidence Synthesis fellowship.

## Supporting information

supmat.

## Endnotes

1 Members include: Bedford Row Family Project, Care After Prison, Fusion Community Prison Link, New Beginnings, Childhood Development Initiative, Travellers in Prison Initiative, Dillon's Cross Project, Children's Rights Alliance, as well as researchers from University College Cork, Maynooth University, Dublin City University and the University of Limerick.
