# Supplementary material for: Protocol: The Impact of Parental Incarceration on Families Affected: An Evidence and Gap Map: A Systematic Review
Source: Campbell Syst Rev. 2025 Aug 17;21(3):e70055. doi: 10.1002/cl2.70055 (PMC12358683; doi:10.1002/cl2.70055)
Supplement: Supplementary file 1 — supmat. [file CL2-21-e70055-s001.docx]

**Appendices**

*Appendices should be submitted as* [*supplementary material*](https://authorservices.wiley.com/author-resources/Journal-Authors/Prepare/manuscript-preparation-guidelines.html/supporting-information.html)*.*

**1 Search strategies:**

Table 2 Table 2 APA Psych Articles search conducted 05/09/2022

| **Step** | **Search Fields** | **Search Syntax** |  |
| --- | --- | --- | --- |
| 1 | Title | (child* OR adolesce* OR teen* OR youth* OR young* OR minor* OR kid* OR student* OR school* OR pube* OR paren* OR pater* OR father* OR dad* OR mater* OR mother* OR mum* OR mom* OR pregn* OR famil* OR son* OR daughter*) | 37,564 |
| 2 | Abstract | (child* OR adolesce* OR teen* OR youth* OR young* OR minor* OR kid* OR student* OR school* OR pube* OR paren* OR pater* OR father* OR dad* OR mater* OR mother* OR mum* OR mom* OR pregn* OR famil* OR son* OR daughter*) | 87,558 |
| 3 | Keywords | (child* OR adolesce* OR teen* OR youth* OR young* OR minor* OR kid* OR student* OR school* OR pube* OR paren* OR pater* OR father* OR dad* OR mater* OR mother* OR mum* OR mom* OR pregn* OR famil* OR son* OR daughter*) | 52,710 |
| 4 | Subject or Indexing Terms | (child* OR adolesce* OR teen* OR youth* OR young* OR minor* OR kid* OR student* OR school* OR pube* OR paren* OR pater* OR father* OR dad* OR mater* OR mother* OR mum* OR mom* OR pregn* OR famil* OR son* OR daughter*) | 53,323 |
| 5 | - | 1 OR 2 OR 3 OR 4 | 110,174 |
| 6 | Title | prison* OR incarcerat* OR imprison* OR jail* OR  gaol* OR correction* OR offend* OR detain* | 4,593 |
| 7 | Abstract | prison* OR incarcerat* OR imprison* OR jail* OR  gaol* OR correction* OR offend* OR detain* | 5,355 |
| 8 | Keywords | prison* OR incarcerat* OR imprison* OR jail* OR  gaol* OR correction* OR offend* OR detain* | 1,763 |
| 9 | Subject or Indexing Terms | prison* OR incarcerat* OR imprison* OR jail* OR  gaol* OR correction* OR offend* OR detain* | 2,573 |
| 10 | - | 6 OR 7 OR 8 OR 9 | 8,045 |
| 11 | - | 5 AND 10 | 3,816 |

## 
